# A Systematic Review on the Association between Obesity and Mood Disorders and the Role of Gut Microbiota

**DOI:** 10.3390/metabo13040488

**Published:** 2023-03-29

**Authors:** Swati Sagarika Panda, Akankshya Nayak, Srishti Shah, Palok Aich

**Affiliations:** 1 School of Biological Sciences, National Institute of Science Education and Research (NISER), Jatni 752050, India; 2 Homi Bhabha National Institute, Training School Complex, Mumbai 400094, India

**Keywords:** mental disorders, mood disorders, obesity, gut–microbiota, metabolites

## Abstract

Obesity is a complex health condition that increases the susceptibility to developing cardiovascular diseases, diabetes, and numerous other metabolic health issues. The effect of obesity is not just limited to the conditions mentioned above; it is also seen to have a profound impact on the patient’s mental state, leading to the onset of various mental disorders, particularly mood disorders. Therefore, it is necessary to understand the mechanism underlying the crosstalk between obesity and mental disorders. The gut microbiota is vital in regulating and maintaining host physiology, including metabolism and neuronal circuits. Because of this newly developed understanding of gut microbiota role, here we evaluated the published diverse information to summarize the achievement in the field. In this review, we gave an overview of the association between obesity, mental disorders, and the role of gut microbiota there. Further new guidelines and experimental tools are necessary to understand the microbial contribution to regulate a balanced healthy life.

## 1. Introduction

Metabolic syndrome is characterized by a multifactorial condition that gives rise to health-associated risk factors [1]. These risk factors involve atherogenic dyslipidemia, hypertension, and insulin resistance. Metabolic syndrome is predominantly driven by obesity [2,3]. The increase in obesity prevalence has become a global problem across all age groups. According to the WHO report, obesity has increased nearly thrice in the last 50 years. Therefore, it is not surprising that obesity is a leading cause of mortality among noncommunicable diseases (WHO report, 2021), [4].

Patients who are obese commonly encounter emotional stress, which can lead to mood disorders (MDs), including depression and anxiety [5,6,7]. Around 581 million people are affected by MDs (WHO report, 2022), but MDs are still largely ignored [8]. Both children and adults have experienced a rise in the number of MD cases linked to obesity [9,10]. It has been reported that there is a 37% higher chance of being obese in MDs patients while there is an 18% higher chance of being depressed in obese patients, which suggests that a bidirectional association may exist between obesity and MDs [11]. Although there has been extensive research on this subject, the mechanism behind this association is still unknown. Previously, research in animal models has suggested a positive correlation between obesity and mood disorders. Studies reveal that, in animal models like mice, high-fat diet-induced obesity evokes mental conditions like anxiety and depression and can even lead to reduced autophagy and increased neuroinflammation [12]. On the other hand, exposure to stressors also affects the emotional brain and eating habits, which might lead to obesity [13,14]. Similarly, there has been some progress in this research topic with human subjects. While most of the articles suggest a positive link between the two conditions, others suggest that the correlation is inconclusive [15,16,17,18]. Additionally, combining the effect of obesity and MDs on health and lifestyle is more severe than the impact of either condition alone [19]. Therefore, it is crucial to understand the link between mood disorders, like anxiety and depression, and obesity to protect the population worldwide.

Obesity is defined as excessive fat accumulation, while depression is associated mainly with brain function. Studies have revealed that changes in the brain structure have been observed in the obese population [20,21,22,23]. Thus, studying the adipose and brain communication or the adipose–brain axis is critical to understanding the underlying mechanism involved in this comorbidity. The trillions of microbes that live inside our gastrointestinal tract, known as gut microbes, are essential for regulating the adipose–brain axis. The homeostatic regulation of the adipose–brain axis by gut microbes establishes the gut–adipose–brain (GAB) axis [24,25,26]. Thus, a healthy microbiota is essential to maintain inter-organ communication, leading to a healthy life. This large population of gut microbiota can interact with the host mainly through secreting various metabolites. By doing so, they are able to control different organs that are present outside the gut. Through this inter-organ communication, microbiota play a massive role in coordinating the metabolic, immune, and nervous system of the host [27,28,29,30,31].

As the microbiota plays a role in energy intake and expenditure, dysbiosis in the microbiota composition could cause inefficient energy utilization and lead to obesity [32,33]. Lean and obese individuals have different gut microbiota compositions, which suggests that gut microbiota may have a role in obesity. Further studies on animal models and the human population have been done to observe the gut microbiota association in obesity. Research on animal models showed that, among other phyla, an abundance of Bacteroidetes and Firmicutes plays a significant role in obesity. However, studies on the human population are still inconclusive [34,35,36].

The gut–brain axis has attracted researchers, as it plays a crucial role in maintaining physical and mental health. Neuro, immune, and hormonal circuits are three major pathways through which bidirectional communication between the gut and brain occurs [37,38]. Any perturbation in the gut microbiota composition could affect the gut–brain axis or, in other words, the mental and physical health of the host. Alteration in the Bacteroidetes and Firmicutes abundance demonstrates a significant role in regulating mental health, which is also supported by their respective genus level data [39,40]. This approach demonstrates the significance of the microbiota role [41]. Most studies investigating the gut–brain axis are limited to an animal model [42]. Few studies have described the human population’s association between gut microbes and MDs. Compared to healthy people, patients with depression and anxiety exhibit a marked change in microbiome composition [32,43]. The reports addressing the underlying mechanism are minimal. Therefore, it needs more investigation within the human population. Since the relationship between obesity and mood disorders has been established, revealing the gut–adipose–brain axis communication is essential for solving this growing issue.

In the current systematic review, we first discussed how the occurrence of metabolic syndrome and depression are related to each other. As Bacteroidetes and Firmicutes are major populated microbes in the human intestine, we tried to explain how these two phyla change in the obese patient. Similarly, we also checked the change in the Bacteroidetes and Firmicutes abundance in the patients suffering from MDs. At last, after doing the meta-analysis, we concluded how these two major phyla regulate the gut–adipose–brain axis, which helps in the onset of both obesity and metabolic syndrome.

## 2. Materials and Methods

### 2.1. Literature Survey

We looked for relevant articles that discussed the relationship between obesity and MDs, obesity and gut microbiota, and MDs and gut microbiota using key terms like “obesity”, “Metabolic syndrome”, “Depression”, “Anxiety”, “depressive disorder”, “anxiety disorder”, “Gut–microbiota”, “Bacteroidetes”, and “Firmicutes”. This comprehensive search was carried out in Google Scholar and PubMed. We also manually looked through several pertinent articles using the bibliography during our literature search.

### 2.2. Selection Criteria

The articles found from the literature survey were carefully reviewed to assess their relevance and importance in connection with our study. The studies that were finally shortlisted for this study met the following criteria: (1) The authors of the literature must include the case-control group in their studies. (2) The literature was written only in English. (3) The publication was based on a study of the human population. (4) Included articles were published before January 2023. (5) For the obesity measurement, only those studies that quantified obesity based on the body mass index (BMI) data were considered. (6) For the MDs measurement, we included publications that used quantitative methods to measure depression and anxiety. (7) For the information regarding the gut microbiota, only those publications that have done 16s rRNA sequencing of the stool sample were chosen. Papers that used RT-PCR to determine the bacterial abundance in the stool were not taken into account. (8) Manuscripts that did not provide information on the microbiota at the phylum level were excluded.

### 2.3. Data Extraction

**Figure 1:**
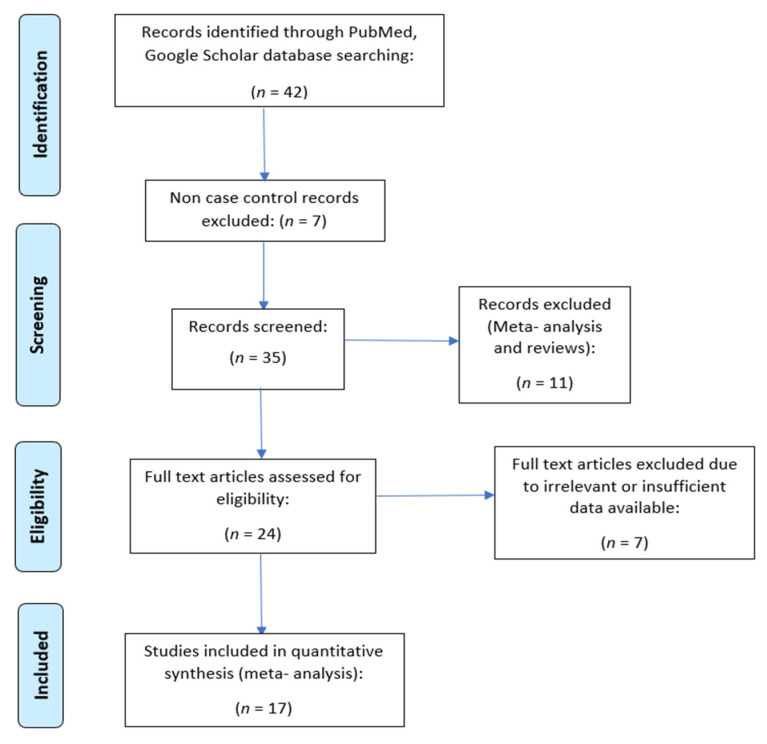
Prisma flowchart depicting the studies selected.

A total of 127 articles were reviewed for our study which included relevant studies for gut microbiota and obesity, gut microbiota and MDs, and obesity and MDs relationship. Out of these 127 studies, 39 studies were dropped since they were non-case-control studies. Another 34 articles were excluded, since they were either meta-analyses or reviews. Out of the remaining 54 articles, another 17 were rejected since they had insufficient or inadequate information regarding the said topic. We ended up with 37 articles out of which 13 studies dealt with the association of MDs with obesity, 11 studies dealt with gut microbiota and obesity, and the remaining 13 studies studied the relationship of gut microbiota with MDs. A flow diagram of included and excluded studies in the meta-analysis is shown in Figure 1. It was designed as per the PRISMA (Preferred Reporting Items for Systematic Reviews and Meta-Analysis) guidelines [Prospero registration number: CRD42023401572]. We extracted the following information from the selected manuscripts: name of the first author, year of publication, criteria of MD evaluation, BMI range for obesity for individual studies, and no. of subjects.

### 2.4. Statistical Analysis

Following the identification of relevant literature, we conducted a meta-analysis. The odds ratio (OR) of the individual studies, the cumulative odds ratio, and the 95% confidence interval were calculated using MedCalc software https://www.medcalc.org (accessed on 2 February 2023). These values were eventually used to determine the association between obesity and gut microbiota, obesity and MDs, and MDs and gut microbiota. We next generated forest plots using the OR values. We also used funnel plots to assess publication bias. It was further estimated with the help of Egger’s test, where the *p*-value of less than 0.05 indicates a significant publication bias.

## 3. Results

### 3.1. Obesity and Mood Disorders

Population studies were retrieved using “PubMed” and “Google Scholar” search engines and were screened using the criteria mentioned in the Prisma graph. Post-screening, 13 papers (a total of 536,185 participants) were selected to investigate the association between obesity and mood disorders like anxiety and depression. Of these 13 studies, 11 were cross-sectional, and 2 were longitudinal.

The standard International Classification System was used where subjects with BMI > 30 were considered obese while those with BMI ≤ 30 were deemed to be non-obese. Among the selected papers, three papers used Composite International Diagnostic Interview (CIDI), four used the Diagnostic and Statistical Manual of Mental Disorders (DSM), one used the WHO composite, two used the Hospital Anxiety and Depression Scale (HADS), one used the Munich-composite, one used Patient Health Questionnaire (PHQ) and one used the International Classification of Diseases (ICD) questionnaires as a measure of Mood Disorder. For each study, the odds of being stressed under an obese condition were calculated with a 95% confidence interval.

All the studies had an odds ratio of more than 1 (Table 1), and the class intervals of all the studies, except the analysis by Hadi et al. [44], do not contain 1 within the 95% confidence interval.

**Figure 2:**
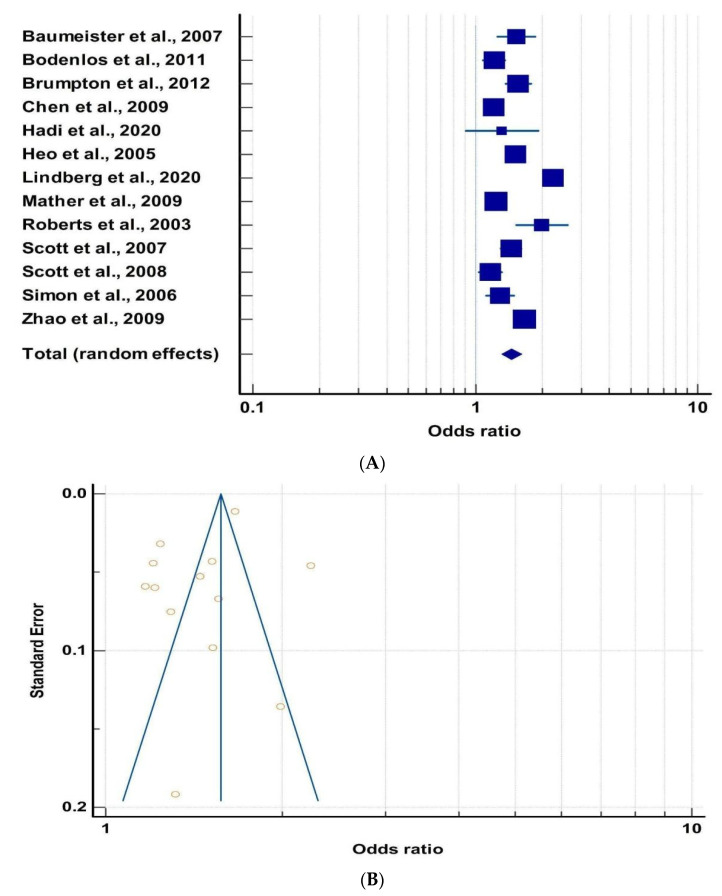
(**A**) Forest plot between obesity and mood disorders [9,44,45,46,47,48,49,50,51,52,53,54,55]. The box and the horizontal lines represent study specific OR and 95% CI, respectively. The size of the box is an indicator of the weightage of that study. Here, odds ratio greater than 1 suggest the odds of mood disorders are higher in obese people in comparison to normal controls. (**B**) Funnel plot of obesity and mood disorders for publication bias.

After calculating the Odds Ratio and 95% Confidence Interval, the studies were subjected to Forest Plot (Figure 2A) using MedCalc to check the consistency of the studies. The Forest Plot of these studies revealed that most of the studies were consistent and clustered around OR = 1.5. Due to a lack of consistency, we discarded three studies by Lindberg et al. [50], Roberts et al. [52], and Hadi et al. [48] from further analysis. The cumulative odds ratio was found to be 1.46, indicating a positive correlation between obesity and mood disorders. Further, a Funnel Plot between Standard Error and Odds Ratio was plotted (Figure 2B) in order to check for publication bias and heterogeneity. Seven out of thirteen studies did not lie within the funnel suggesting publication bias and/or heterogeneity among the studies. In order to quantify this, we performed Egger’s test. The *p*-value of Egger’s test was found to be 0.1748, suggesting that there was no publication bias. However, the studies showed significant heterogeneity *p* < 0.0001.

### 3.2. Microbiota and Obesity

**Figure 3:**
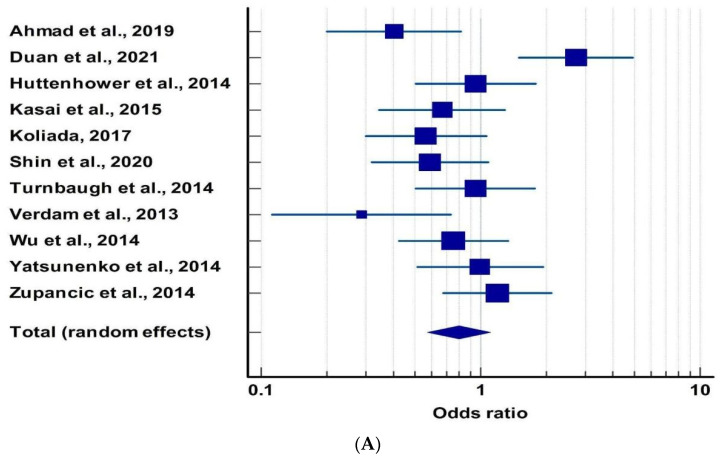

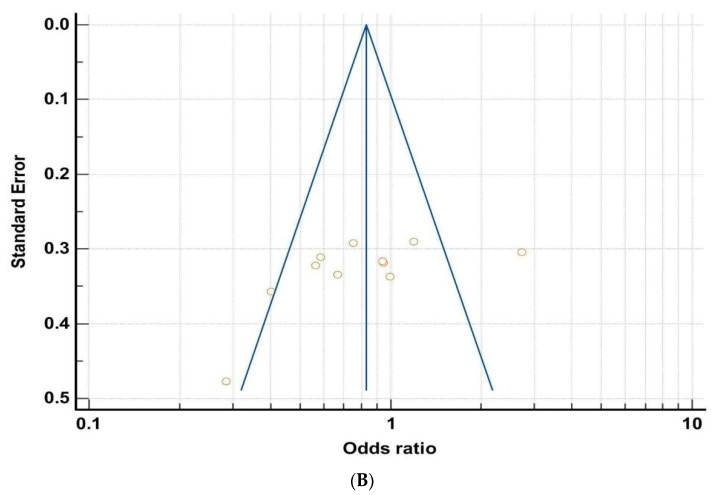
(**A**) Forest plot between obesity and microbiota [56,57,58,59,60,61,62,63,64,65,66]. The box and the horizontal lines represent study specific OR and 95% CI, respectively. The size of the box is an indicator of the weightage of that particular study. Here, odds ratio less than 1 suggest the relative abundance of Firmicutes is higher in obese people. (**B**) Funnel plot of obesity and microbiota for publication bias.

A total of 11 articles were selected to study the correlation of gut microbiota with obesity. Data on the percentage abundance of Bacteroidetes and Firmicutes in obese and normal people were collected from these manuscripts. The odds ratio with a 95% confidence interval was calculated for each of these studies (Table 2). The calculated odds ratio and 95% confidence intervals (CI) were then used to get the forest plot (Figure 3A) using the software MedCalc.

The BMI range for the distinction between obese and normal people varied in most studies. Nine out of eleven studies took a BMI of greater than 30 kg/m^2^ as obese and a BMI of less than or equal to 25 as normal. However, a BMI greater than or equal to 25 and BMI between 18.5–24.9 kg/m^2^ were considered to be obese and normal, respectively, in Kasai’s 2015 study [59]. The study by Shin et al. (2020) [61] also considered a BMI greater than or equal to 25 kg/m^2^ obese.

All the studies collected fecal samples from their respective volunteers (obese and normal). They used this fecal sample for further analysis, which included genomic DNA extraction and PCR amplification, followed by 16s rRNA gene sequencing.

From the forest plot, we observed that not all the studies followed a similar trend to their inferences. Ten out of eleven studies showed clustering near the odds ratio value of (1.428)^−1^. Only one of the studies, Duan (2021) [57], fell out of the cut-off range. Studies excluding Zupancic et al. (2014) [66], Yatsunenko et al. (2014) [65], Wu et al. (2014) [64], Turnbaugh et al. (2014) [62], and Kasai et al. (2015) [59] have had higher contributions to our study. Out of eleven studies, three studies had an odds ratio close to 1, which indicates no potential relationship between the abundance of firmicutes and bacteroidetes and obesity. Six studies had an odds ratio of less than 1, which supports the notion of an increase in Firmicutes in the case of obesity, whereas the remaining two studies with an odds ratio greater than 1 state otherwise. The Funnel plot (Figure 3B) was used to check for publication bias and heterogeneity. The *p*-value of Egger’s test came out to be 0.6328, which suggests that there is no publication bias, but the studies showed significant heterogeneity with a *p*-value of 0.0009 and a value of 0.0009.

### 3.3. Microbiota and Mood Disorders

We took thirteen studies into account to establish a connection between gut microbiota and mood disorders (Table 3). We only addressed research that used information about the human population. Out of thirteen studies, four studies used both the DSM and HAMD rating scale. One investigation used the Beck depression and the Beck anxiety inventory (BDI, BAI) alone, while other studies used DSM along with BDI and BAI. Three studies used MINI following HAMD, DSM, or Hamilton anxiety (HAMA). Guo et al. used HAMD and HAMA to assess depression and anxiety, while Jiang et al. used HAMD and MADRS. One investigation used the HAMD and Montreal Cognitive Assessment (MoCA). Hu et al. used MINI, DSM, HAMD, and MADRS.

All the studies employed 16s rRNA sequencing of fecal samples to describe the microbiota status. Most research described the gut microbiota composition in patients with MDD, although Guo et al. examined the composition in patients with MDD who also had irritable bowel syndrome [68]. Except for one study, which exclusively examined the gut microbiota in anxiety patients [76], all studies detailed the gut microbiota composition in depressed patients.

**Figure 4:**
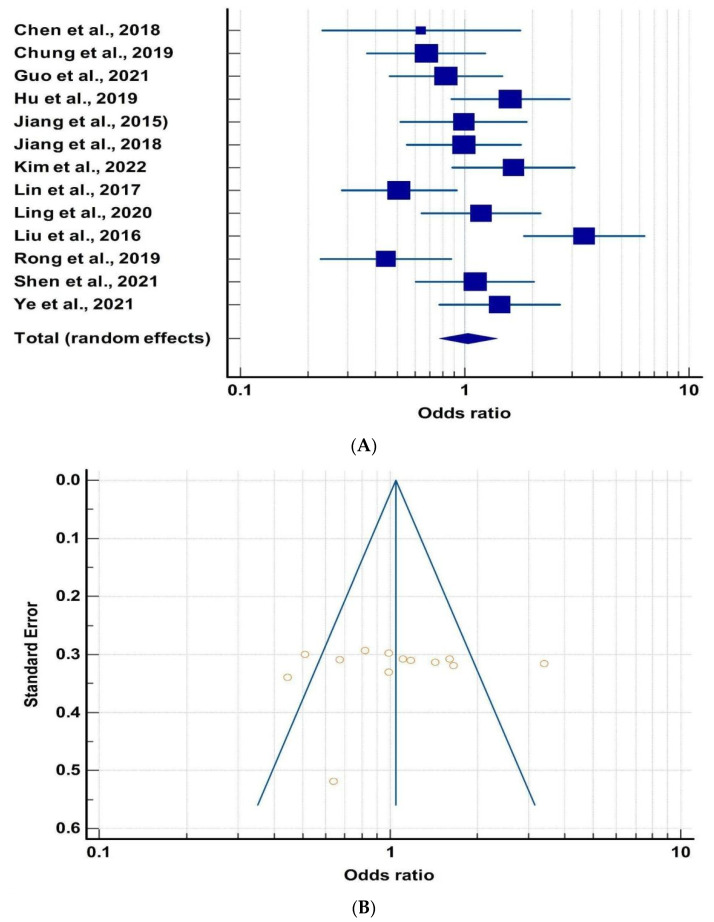
(**A**) Forest plot between mood disorders and microbiota [67,68,69,70,71,72,73,74,75,76,77,78,79]. The box and the horizontal lines represent study specific OR and 95% CI, respectively. The size of the box is an indicator of the weightage of that particular study. Here, odds ratio greater than 1 suggest that the relative abundance of Bacteroidetes is higher in people with mood disorders. (**B**) Funnel plot of mood disorders and microbiota for publication bias.

We calculated the odds ratio for each of the thirteen studies (Table 3). The calculation was based on the odds of having more Bacteroidetes under MDs. All the studies, except one [74], came in the 95% confidence interval level range. Therefore, for further analysis, we dropped that study. Out of thirteen studies, seven studies, i.e., Chen et al., 2018, Chung et al., 2019, Guo et al., 2021, Jiang et al., 2015, Jiang et al., 2018, Lin et al., 2017, Rong et al., 2019 showed the odd ratio value 0.64, 0.67, 0.82, 0.98, 0.99, 0.51, 0.44, respectively [67,68,69,70,71,76,79]. In contrast, Hu et al., 2019, Kim et al., 2022, Ling et al., 2020, Shen et al., 2021, and Ye et al., 2021 showed odd ratios of 1.6, 1.65, 1.18, 1.11, and 1.42 [72,73,74,75,77,78]. The Forest Plot of these studies was done using MedCalc software (Figure 4A). The pooled odds ratio for a high relative abundance of Bacteroidetes in MD patients was 1.037, with a CI (95%) from 0.770 to 1.397. The Funnel Plot (Figure 4B) with Egger’s test gave a *p*-value of 0.6328, indicating no publication bias. However, it showed significant heterogeneity with a *p*-value of 0.0005.

## 4. Discussion

This study uses a systematic literature review and subsequent meta-analysis to understand the relationship between obesity and mood disorders. The results of this study are consistent with the existing literature, which suggests that the chances of mood disorders are higher in obese patients and vice versa. There have been several studies that have attempted to understand the association between obesity and mood disorders. While the underlying mechanism is still undiscovered, the relationship between the two has been suggested to be bidirectional. Evidence suggests that mood disorders like anxiety and depression can have negative consequences, such as reduced eating regulation [80]. In such a case, the body might become less sensitive to hunger cues and lose the ability to distinguish between hunger and the mere emotional desire to eat [81]. This might result in emotional eating, where eating becomes an emotional response to any kind of adverse situation [82,83,84], which might eventually lead to overeating, weight gain, and subsequent obesity [85,86]. In the case of stressed individuals, dysregulation in the hypothalamus-pituitary-adrenal axis might cause weight gain [87,88,89]. Additionally, chronic conditions like asthma that are often associated with anxiety might negatively affect proper body functioning and reduce physical activity, resulting in conditions like obesity [88]. Conversely, obesity might also lead to severe mood disorders. One of the most common ideologies embedded in modern society is the ‘thin ideal’ [90], which increases the prevalence of body shaming, leading to lower self-esteem in overweight and obese people. Such stigmatizations negatively impact individuals’ emotional and psychological health [91], leading to mood disorders like anxiety and depression. Apart from these, other factors like genetics [88,92,93] and hormonal imbalance [87,88,89,94] might act as a linker between the two conditions. Studies have shown that the risks of such diseases are more in the case of women than in men [95,96]. Apart from the physiological differences between both sexes, the differences in societal pressure might also lead to such variance among the sexes. Our study also shows that the degree of association between the two conditions varies. This variation may be due to sex, age, occupation, or socio-economic status.

In recent years, there has been substantial scientific interest and advances in gut microbiota and obesity. Marked differences in the composition and function of the microbiota have been reported in the case of obese and normal subjects [97]. It has been seen that this changed microbiota favors energy-harvesting, promoting obesity [98]. In addition, gut microbiota also influences appetite, chronic inflammation, circadian rhythm, and fat storage, which contribute to obesity [99]. Some of the major phyla of bacteria that compose the gut microbiota are Firmicutes, Bacteroidetes, Proteobacteria, Verrucomicrobia, Actinobacteria, and Fusobacteria, with a predominance of Firmicutes (Gram-positive) and Bacteroidetes (Gram-negative) [100]. Both these phyla are involved in carbohydrate metabolism, with Bacteroidetes also being active in energy production and amino acid transport and metabolism [101]. Since Bacteroidetes and Firmicutes play such a crucial role in metabolism and host homeostasis, they are considered biomarkers for obesity [99]. A change in the abundance of Firmicutes to Bacteroidetes can signify dysbiosis in the gut microbiota, which could lead to different disorders. Studies suggest that a relative increase in Firmicutes compared to Bacteroidetes indicates obesity, whereas its reciprocal can be a sign of Inflammatory Bowel diseases (IBD) [102]. Our study here also focuses mainly on these two phyla. Our results suggest that an increase in Firmicutes and a decrease in Bacteroidetes are observed in obese patients compared to normal subjects. This observation is consistent with most of the work taken up for this study. A study conducted on Ukrainian patients showed a gradual increase and decrease in the abundance of Firmicutes and Bacteroidetes, respectively, with rising BMI values [60]. These results are also supported by a few other studies [59,103,104]. In contrast, Duan et al. [57] showed that in the case of obesity, there was a decrease in the abundance of Firmicutes and an increase in Bacteroidetes. This result in obese subjects is consistent with the findings of Schiwiert et al. [105]; however, there was no significant change in Bacteroidetes population, but a decreased level of Firmicutes was seen. This conclusion is also supported by the study performed by Verdam et al. [63]. Some studies have pointed out the difficulty of associating the relative abundance of Firmicutes and Bacteroidetes with obesity as the gut microbiota is highly dynamic and is influenced by various factors [106,107]. The odds ratio from different studies varies to a great extent, which may be due to the variation in age, gender, race, ethnicity, and environmental conditions. As mentioned earlier, Bacteroidetes and Firmicutes participate in various metabolic reactions and, as a result, produce different metabolites. Therefore, any dysbiosis in the Firmicutes and Bacteroidetes level in gut microbiota is reflected in the microbiota-derived metabolites. One such metabolite, Trimethylamine N- oxide (TMAO), has been seen to increase in cases of obesity, and one of the TMAO-producing bacteria belongs to Firmicutes phyla. The observed increase in Firmicutes percentage in the case of obese subjects might lead to this rise in the TMAO, as seen in the obese group [108]. In addition to this, literature also suggests that changes in the circadian rhythm are linked to a plethora of metabolic diseases including obesity. The link between the two is attributed to the perturbation of gut flora due to fluctuations in the circadian clock. It is also observed that microbial transfer from healthy individuals restores the normal conditions in case of circadian rhythm disruptions-induced metabolic diseases [109]. This shows us the crucial role the gut microbiota plays in the development of metabolic diseases, especially obesity. Further investigation into these observed changes could lead to some answers in order to tackle this widespread metabolic syndrome—obesity.

On the other hand, the gut flora is one of the significant regulators of brain function [110,111]. It communicates with the brain primarily through metabolites, the vagus nerve, the hypothalamic-pituitary-adrenal axis (HPA), and the immune system [112,113]. Therefore, it is crucial to investigate the gut microbiota composition in MDs patients. It has been noted that the two dominant phyla in the human population are Bacteroidetes and Firmicutes [114]. There is a variation in the gut microbiota structure between the MDs population and the healthy control population. Research revealed a decrease in Firmicutes and a rise in Bacteroidetes in patients with MDs, whereas the other two investigations revealed the opposite outcome [39,115,116]. Therefore, inconsistencies exist in the relative abundance of Bacteroidetes and Firmicutes in patients suffering from MDs [111,117]. We conducted a meta-analysis to learn more about the crosstalk between Bacteroidetes and Firmicutes in MD patients. Out of thirteen studies, seven had an odds ratio below one, suggesting that the Bacteroidetes may have a more profound impact on MD patients [67,68,69,70,71,76,79]. Other five studies showed an odds ratio above one, indicating that MD patients had a higher relative abundance of Firmicutes in comparison to the Bacteroidetes [72,73,74,75,77,78]. However, the pooled odds ratio gave an inconclusive result about the respective phyla abundance in MDs population. Apart from this, the studies showed significant heterogeneity, suggesting more variation among the studies than by chance alone. This might be due to varied factors like sex, age, ethnicity, socio-economic status, diet, and physical activity, among others. This study did not investigate the changes observed in the lower taxonomic level under Bacteroidetes and Firmicutes phylum. The literature suggests that these two phyla also show an irregular pattern at the lower taxonomic level [9,112,118]. The possible reasons may include a small population size, different age groups in the population, the sensitivity of gut microbiota which could respond to even minor changes in the conditions, and the health condition of the control population, etc. We should switch from 16S rRNA sequencing to complete metagenome analysis to avoid these problems. We also have to increase the population size and study the timeline to know the long-term changes in gut flora and its impact on host health.

One of the principal limitations of this study is the number of studies included is limited, and most of the data available are from developed nations; hence, the generalizability of this correlational study to developing countries is questionable. For example, low socio-economic conditions in a developing country might restrict the citizens from lavish spending on food and increased labor, which might affect the chances of obesity. Apart from this, there are different questionnaires/interviews to assess mood health conditions, and the thresholds for categorization might vary among them. The studies can be easily misguided in case of any wrong information provided by the participants. Anxiety and depression symptoms qualify as clinical conditions only when they significantly disrupt the proper functioning of the body. Thus, the concept of mood disorder is contextual.

## 5. Conclusions

This systematic review and meta-analysis supports the previous findings that suggest that obesity and mood disorders are positively correlated, and that the relative abundance of Bacteroidetes and Firmicutes might play a role in the diseased conditions. While the majority of the data suggest that the relative abundance of Firmicutes is higher in the case of obese people, the significance of the studies still needs to be validated with a larger sample size. Meanwhile, in the case of mood disorders, it is difficult to comment on the relative abundance of the two species. As a future perspective of this study, we need to include more samples to establish a stronger relationship between the relative abundance of specific microbiota in both the diseased conditions. Once such a microbial population is identified, it will facilitate us to establish a mechanism-based correlation between obesity and mood disorders with the help of the metabolites derived from those specific microbial populations.

## Figures and Tables

**Table 1:** Population data of included studies.

Publication	Reference	Criteria for MD Evaluation	No. of Subjects (N)	OR	95% CI	
					Lower CI	Upper CI
Baumeister et al., 2007	[45]	Munich composite	2955	1.53	1.26	1.85
Bodenlos et al., 2011	[46]	CIDI	10,899	1.22	1.08	1.37
Brumpton et al., 2012	[47]	Hospital Anxiety and Depression scale	44,076	1.56	1.37	1.78
Chen et al., 2009	[48]	CIDI	59,652	1.21	1.1	1.31
Hadi et. al., 2020	[44]	HAMD	614	1.32	0.9	1.91
Heo et al., 2005	[49]	DSM	34,702	1.52	1.4	1.66
Lindberg et al., 2020	[50]	ICD-10, ATC, etc.	72,570	2.24	2.05	2.45
Mather et al., 2009	[51]	DSM	34,900	1.24	1.16	1.32
Roberts et al., 2003	[52]	DSM	3772	1.99	1.52	2.59
Scott et al., 2008	[53]	DSM	12,738	1.45	1.31	1.61
Scott et al., 2008	[9]	CIDI 3.0	73,135	1.17	1.04	1.31
Simon et al., 2006	[54]	WHO Composite	9125	1.29	1.12	1.5
Zhao et al., 2009	[55]	PHQ-8	177,047	1.67	1.63	1.7
Cumulative Odds: 1.46						

OR: Odds Ratio CI: Confidence Interval.

**Table 2:** Microbiota and obesity data from the considered studies.

Publication	Reference	BMI Range (kg/m^2^)		No. of Subjects (N)	OR	95% CI	
		Obese	Normal			Lower CI	Upper CI
Ahmad et al., 2019	[56]	32.4 + −3.6	22.08 + −3.1	60	0.40	0.19	0.81
Duan et al., 2021	[57]	>30	<25	42	2.72	1.49	4.94
Huttenhower et al., 2014	[58]	>30	<25	N/A	0.95	0.51	1.77
Kasai et al., 2015	[59]	≥25	18.5–25	56	0.67	0.35	1.28
Koliada et al., 2017	[60]	>30	18.5–24.9	38	0.56	0.29	1.06
Shin et al., 2020	[61]	18.5–25	≥25	46	0.58	0.32	1.08
Turnbaugh et al., 2014	[62]	>30	<25	154	0.94	0.51	1.76
Verdam et al., 2013	[63]	>30	<25	28	0.29	0.11	0.73
Wu et al., 2014	[64]	>30	<25	N/A	0.75	0.42	1.33
Yatsunenko et al., 2014	[65]	>30	<25	N/A	0.99	0.51	1.93
Zupancic et al., 2014	[66]	>30	<25	310	1.19	0.68	2.10
Cumulative Odds: 0.797							

OR: Odds Ratio, CI: Confidence Interval, N/A: Not Available.

**Table 3:** Microbiota and MD data from the considered studies.

Publication	Reference	Criteria for MDs Evaluation	No. of Subjects (N)	OR	95% CI	
					Lower CI	Upper CI
Chen et al., 2018	[67]	DSM, HAMD	48	0.64	0.23	1.76
Chung et al., 2019	[68]	DSM, BAI, BDI	73	0.67	0.36	1.23
Guo et al., 2021	[69]	HAMD, HAMA	18	0.82	0.46	1.46
Hu et al., 2019	[70]	MINI, DSM, HAMD, MADRS	97	1.6	0.87	2.92
Jiang et al., 2015	[71]	HAMD, MADRS	59	0.98	0.51	1.89
Jiang et al., 2018	[72]	MINI, HAMA	76	0.99	0.55	2.77
Kim et al., 2022	[73]	BAI, BDI	39	1.65	0.88	3.09
Lin et al., 2017	[74]	DSM, HAMD	20	0.51	0.28	0.91
Ling et al., 2020	[75]	HAMD, MoCA	66	1.18	0.64	2.16
Liu et al., 2017	[76]	MINI, DSM	35	3.4	1.83	6.31
Rong et al., 2019	[77]	DSM, HAMD	61	0.44	0.22	0.86
Shen et al., 2021	[78]	MINI, HAMD	60	1.11	0.6	2.03
Ye et al., 2021	[79]	DSM, HAMD	54	1.42	0.77	2.64
Cumulative Odds: 1.03						

OR: Odds Ratio. CI: Confidence Interval.
